# Serum neurofilament light chain levels are associated with early neurological deterioration in minor ischemic stroke

**DOI:** 10.3389/fneur.2023.1096358

**Published:** 2023-03-09

**Authors:** Jie Li, Ping Zhang, Yalan Zhu, Yong Duan, Shan Liu, Jie Fan, Hong Chen, Chun Wang, Xingyang Yi

**Affiliations:** ^1^Department of Neurology, Deyang People's Hospital, Deyang, China; ^2^Department of Neurology, Guanghan People's Hospital, Deyang, China; ^3^Department of Neurology, Zhongjiang People's Hospital, Deyang, China; ^4^Department of Neurology, Deyang Jingyang District Hospital of Traditional Chinese Medicine, Deyang, China; ^5^Department of Neurology, Deyang Hospital of Integrated Traditional Chinese and Western Medicine, Deyang, China

**Keywords:** minor ischemic stroke, neurological deterioration, neurofilament light chain, odds ratio, biomarker

## Abstract

**Objectives:**

Patients with minor ischemic stroke (MIS) frequently suffer from early neurological deterioration (END) and become disabled. Our study aimed to explore the association between serum neurofilament light chain (sNfL) levels and END in patients with MIS.

**Methods:**

We conducted a prospective observational study in patients with MIS [defined as a National Institutes of Health Stroke Scale (NIHSS) score 0–3] admitted within 24 h from the onset of symptoms. sNfL levels were measured at admission. The primary outcome was END, defined as an increase in the NIHSS score by ≥2 points within 5 days after admission. Univariate and multivariate analyses were performed to explore the risk factors associated with END. Stratified analyses and interaction tests were conducted to identify variables that might modify the association between sNfL levels and END.

**Results:**

A total of 152 patients with MIS were enrolled, of which 24 (15.8%) developed END. The median sNfL level was 63.1 [interquartile range (IQR), 51.2–83.4] pg/ml on admission, which was significantly higher than that of 40 age- and sex-matched healthy controls (median 47.6, IQR 40.8–56.1 pg/ml; *p* < 0.001). Patients with MIS with END had a higher level of sNfL (with ND: median 74.1, IQR 59.5–89.8 pg/ml; without END: median 61.2, IQR 50.5–82.2 pg/ml; *p* = 0.026). After adjusting for age, baseline NIHSS score, and potential confounding factors in multivariate analyses, an elevated sNfL level (per 10 pg/mL) was associated with an increased risk of END [odds ratio (OR) 1.35, 95% confidence interval (CI) 1.04–1.77; *p* = 0.027). Stratified analyses and interaction tests demonstrated that the association between sNfL and END did not change by age group, sex, baseline NIHSS score, Fazekas' rating scale, hypertension, diabetes mellitus, intravenous thrombolysis, and dual antiplatelet therapy in patients with MIS (all *p* for interaction > 0.05). END was associated with an increased risk of unfavorable outcomes (modified Rankin scale score ranging from 3 to 6) at 3 months.

**Conclusion:**

Early neurological deterioration is common in minor ischemic stroke and is associated with poor prognosis. The elevated sNfL level was associated with an increased risk of early neurological deterioration in patients with minor ischemic stroke. sNfL might be a promising biomarker candidate that can help to identify patients with minor ischemic stroke at high risk of neurological deterioration, for reaching individual therapeutic decisions in clinical practice.

## Introduction

Minor ischemic stroke (MIS) is fairly common and accounts for about 30% of all strokes (1). Although most patients with MIS have favorable outcomes, a small but significant proportion of individuals suffer neurological deterioration in the early stages of acute ischemic stroke (AIS) and become disabled (2, 3). It also has been demonstrated that early neurological deterioration (END) after ischemic stroke is an independent predictor of poor prognosis (4–6). Several hypotheses have been proposed regarding the mechanisms of END, including the propagation of thrombus *in situ*, inflammation, excitotoxicity, oxidative stress, and cortical spreading depolarization (7). However, until recently, the underlying pathophysiology of END in patients with MIS is still unclear (8). Once END occurs, there are no effective therapies to arrest it. Thus, the early identification and rational prevention of END are essential for this ominous event.

Neuronal damage and loss are the pathological basis of disability caused by cerebral infarction. As a part of the neuronal cytoskeleton that is exclusively expressed in neurons, neurofilaments are suitable candidate biomarkers for neuronal injury (9). When ischemic damage occurs, neurofilament light chain (NfL) protein is released into the extracellular fluid, the cerebrospinal fluid, and to a lower concentration in the peripheral blood (10). With the application of single-molecule array (SiMoA) assays that enabled a sensitive detection of NfL in blood samples, neurofilaments are gaining increasing attention in various neurologic diseases such as traumatic brain injury, multiple sclerosis, dementias, and different neurodegenerative diseases (9). In patients with ischemic stroke, serum NfL (sNfL) levels have been correlated with initial stroke severity assessed by the NIHSS score on admission (11–14). Meanwhile, the baseline NIHSS score has been shown to be a good predictor of the course of END (15–17). Some studies have suggested that baseline sNfL is a valuable biomarker of the functional outcome at 3 months after cerebral infarction (12, 14), but others have reached a different conclusion (11, 13). sNfL has also been shown to be associated with active small vessel disease (18). Until recently, whether sNfL levels are associated with END in patients with MIS has not been elucidated.

Therefore, the current study aimed to explore the potential association of sNfL levels with END in Chinese patients with MIS.

## Methods

### Study design and subjects

Patients with AIS admitted to Deyang People's Hospital were prospectively and consecutively registered from 1 March 2020 to 31 June 2021. Patients with MIS who were admitted within 24 h from the symptom onset and with magnetic resonance diffusion-weighted imaging (DWI) diagnoses of cerebral infarction were eligible for this observational study. MIS was defined as having a National Institutes of Health Stroke Scale (NIHSS) score of ≤3 points at admission (19). All patients received an extensive stroke etiologic workup (computed tomographic angiography or magnetic resonance angiography, color duplex ultrasound, Holter monitoring, echocardiography, and blood sampling) and were routinely followed up after 3 months by telephone interview or by mail. We excluded cases with incomplete hospital records or missing imaging that would prevent complete data collection. We also excluded subjects with a preexisting score of more than 2 on the modified Rankin scale (mRS, a scale of 0 to 6, with 0 indicating no symptoms and 6 indicating death) and lived dependently (20). Meanwhile, cases with comorbid disorders that could lead to neuronal damage, such as traumatic brain injury, multiple sclerosis, dementia, and other neurological diseases were excluded. The study was approved by the Ethics Committee of Deyang People's Hospital (Reference No. 2019-01-142-K01) and was carried out under the principles expressed in the Declaration of Helsinki. Written informed consent was obtained from all patients before they were enrolled. The study described here is registered at http://www.chictr.org/ (unique identifier: ChiCTR2000029902). The date of trial registration was 16 February 2020. All methods in the present study were performed according to relevant guidelines and regulations.

### Data collection

Baseline data on age, sex, onset to admission time, baseline NIHSS score, systolic and diastolic blood pressure on admission, baseline serum glucose, vascular risk factors, and potential stroke etiology were recorded, which has been described in our previous study (21). Results of routine laboratory tests such as triglycerides (TG), total cholesterol (TC), low-density lipoprotein cholesterol (LDL-C), high-density lipoprotein cholesterol (HDL-C), uric acid, fibrinogen, d-dimer, and C-reactive protein (CRP) were also collected. The white matter lesions (WMLs) were visually evaluated by experienced neuroradiologists using the modified Fazekas scale (22, 23). The Fazekas scale is a 4-point rating scale: 0 (no WML), 1 (mild WML), 2 (moderate WML), and 3 (severe WML). White matter changes were divided into two groups: 0–1 (absent or mild) or 2–3 (moderate or severe). In-hospital treatments analyzed in our study included intravenous thrombolysis and antiplatelet therapy. Intravenous thrombolysis was performed according to the Chinese guidelines, which had similar inclusion and exclusion criteria compared with the American guidelines (24, 25). The final treatment decision was made in consultation with the neurologist and the patient's family. Antiplatelet therapies were administered at the physicians' discretion. Patients enrolled in the present study received either (1) aspirin or clopidogrel only or (2) clopidogrel plus aspirin (dual antiplatelet therapy) at admission.

### Measurement of sNfL levels

Whole blood samples (4 ml) were drawn from all patients with MIS at admission, and serum samples were isolated following centrifugation for 20 min at 2,000 g at room temperature. Then, serum samples were stored at −80°C until analysis. At the same time, 40 age- and sex-matched healthy controls were selected, and their serum samples were obtained after their enrollment in the study. Serum NfL (sNfL) concentrations were measured using a SiMoAplatform (Quanterix, Lexington, MA, United States) as described (26). All serum samples were analyzed in duplicates for inter-test validation, and the two results were averaged to determine the mean concentration. The mean intra-assay variability (the coefficient of the variation of concentrations) was <10%, and the inter-assay coefficient of variation was <15%.

### Assessment of clinical outcomes

The primary outcome of the present study was END, which was defined as an increase in NIHSS score by 2 or more points within 5 days after admission, after excluding the hemorrhagic transformation of the brain infarct or a new infarct in another vascular territory (27). Trained neurologists assessed the neurological severity of the patients daily between admission and discharge from the stroke unit. The secondary outcome measures in our study were 3-month death and an unfavorable outcome [defined as having an mRS score of 3–6 (20)].

### Statistical analyses

Continuous variables are presented as mean with standard deviation (SD) or median with interquartile range (IQR), and categorical variables are presented as frequencies with percentages. The normality of data was tested using a Shapiro–Wilk test. Baseline characteristics, laboratory values, and in-hospital treatment were compared between patients with MIS with or without END. The χ^2^ tests or Fisher's exact tests were used for differences in categorical data, while Student's *t*-tests or the Mann–Whitney U-test were used for differences in continuous data. Multivariate logistic regression analysis was performed using the forced entry method, including variables with a *p*-value of <0.1 in univariate analyses, to identify the association between sNfL levels and END in patients with MIS. Then, stratified analyses and interaction tests were conducted to identify variables that might modify the association between sNfL levels and END. All statistical analyses were performed using SPSS v21.0 (IBM, Chicago, IL, USA), the statistical software packages R (http://www.R-project.org, The R Foundation, version 3.4.3), and EmpowerStats (http://www.empowerstats.com, X&Y Solutions, Inc., Boston, MA, USA), which have been described in our previous study (28). A two-sided *p*-value of < 0.05 denoted statistical significance.

## Results

**Figure 1:**
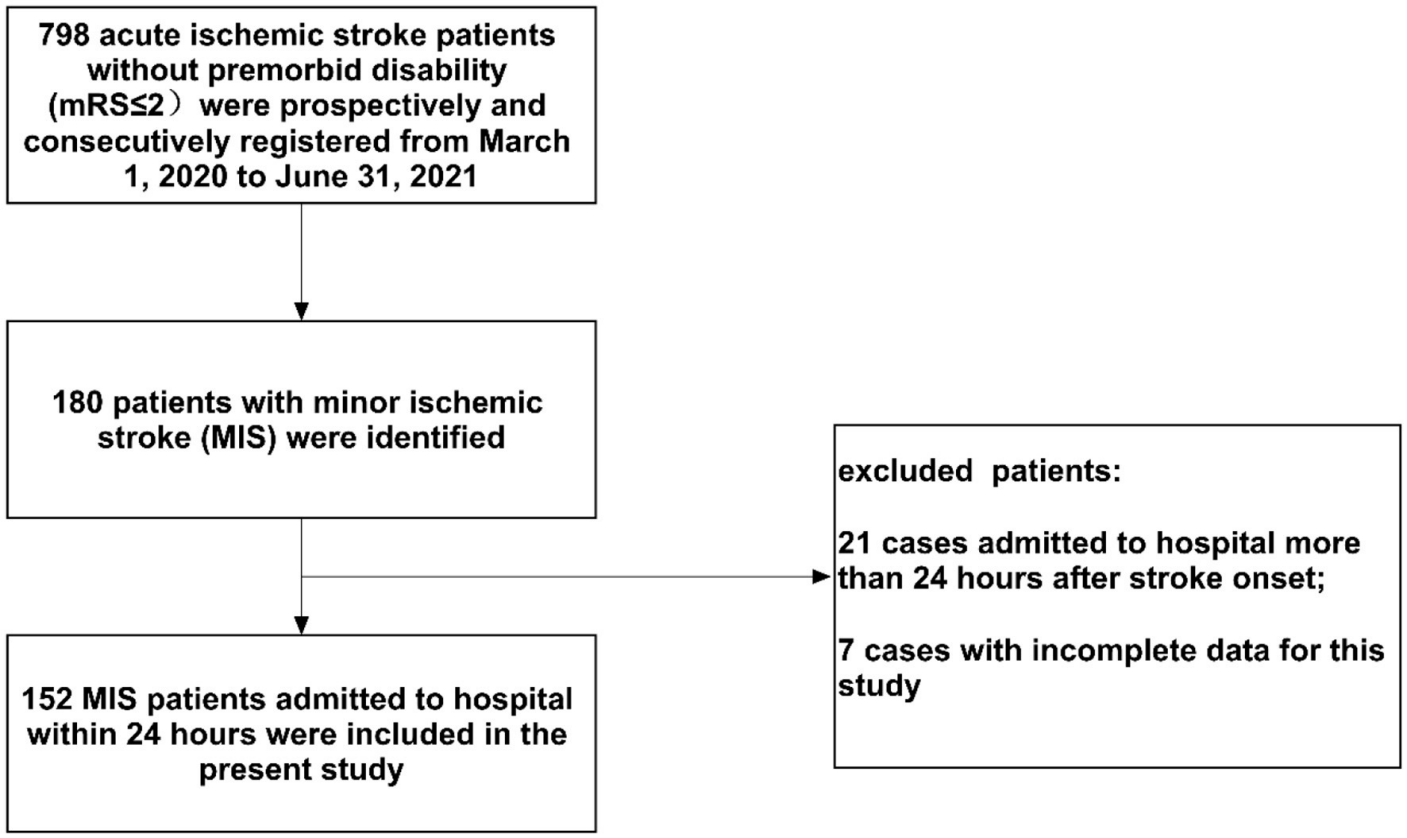
Flow diagram of included and excluded patients.

**Figure 2:**
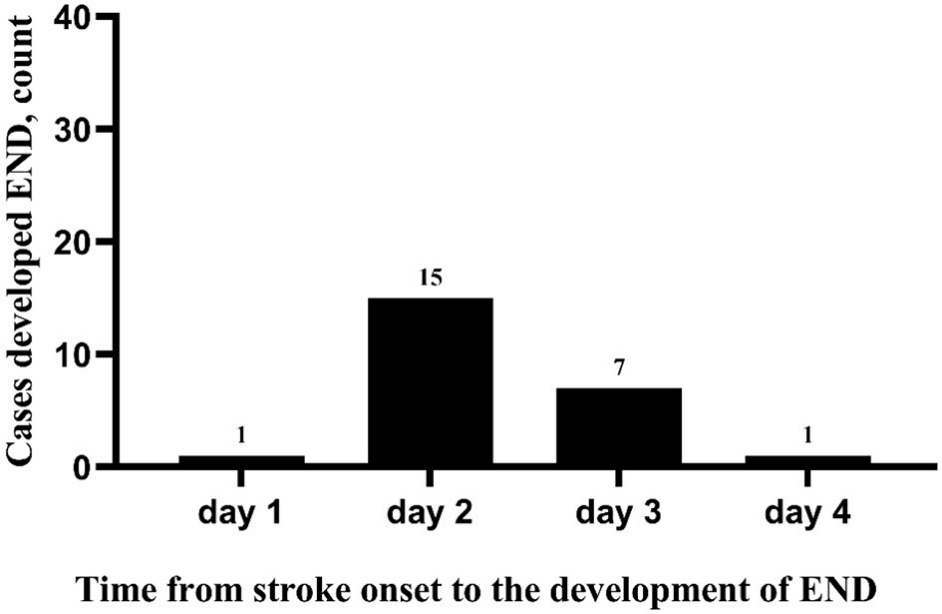
Time from the stroke onset to the development of END.

During the study period, 798 patients with AIS were registered. Of those patients, 152 (19.0%) patients with MIS admitted within 24 h were enrolled in the present study (median baseline NIHSS score: 2, IQR: 1–3). A flow diagram of included and excluded patients is provided in Figure 1. Their age varied from 32 to 95 years (mean age 67.7 and standardized difference 11.1), and 104 (68.4%) were men. The median onset to admission time was 8.5 h (IQR: 3.0–20.8 h). On admission, the median sNfL levels of enrolled patients with MIS were 63.1 pg/ml (IQR: 51.2–83.4 pg/ml). We did not observe a significant correlation between the baseline NIHSS score and sNfL values at hospital admission (Spearman correlation analysis, rho = 0.150, *p* = 0.066) (Supplemental Figure 1). A total of 30 (19.7%) cases were treated with intravenous thrombolysis, and 109 (71.7%) were treated with dual antiplatelet therapy at admission (Table 1). A total of 24 (15.8%) patients experienced END within 5 days after admission. The median time from the stroke onset to the development of END was 48 h (IQR: 30–49.75 h). In 22 out of the 24 patients (91.7%), END was observed on days 2 and 3 after the stroke onset (Figure 2). All enrolled cases completed 3-month follow-up. In total, three (2.0%) patients died, and 10 (6.6%) patients had unfavorable outcomes at 3 months (Table 1).

**Table 1:** Baseline characteristics and clinical outcomes of patients with MIS.

	**MIS (*n* = 152)**
Age, years	67.7 ± 11.1
Sex (male)	104 (68.4)
Onset to admission time, hours	8.5 (3.0–20.8)
Baseline NIHSS score	2 (1–3)
SBP on admission (mm Hg)	157.9 ± 25.4
DBP on admission (mm Hg)	88.7 ± 14.4
Baseline serum glucose (mmol/L)	7.2 (6.0–10.8)
**Risk factors**	
Hypertension	133 (87.5)
Diabetes mellitus	54 (35.5)
Dyslipidemia	52 (34.2)
Coronary heart disease	16 (10.5)
Atrial fibrillation	25 (16.4)
Rheumatic heart disease	4 (2.6)
Gout	6 (3.9)
Current smoking	83 (54.6)
Previous ischemic stroke/TIA	24 (15.8)
Previous ICH	6 (3.9)
**TOAST classification**	
Large-artery atherosclerosis	49 (32.2)
Cardio-embolism	16 (10.5)
Small vessel occlusion	70 (46.1)
Other determined etiology	2 (1.3)
Undetermined etiology	15 (9.9)
Fazekas' rating scale (score 2–3)	52 (34.2)
**Laboratory values**	
Serum NfL (pg/ml)	63.1 (51.2–83.4)
TG (mmol/L)	1.4 (1.0–2.0)
TC (mmol/L)	4.5 ± 0.9
LDL-C (mmol/L)	2.5 ± 0.7
HDL-C (mmol/L)	1.3 ± 0.3
Uric acid (μmol/L)	359.2 (294.7–424.3)
Fibrinogen (mg/dL)	2.4 (2.2–3.0)
D-dimer (mg/dL)	0.39 (0.23–0.72)
CRP (mg/L)	0 (0-1.83)
**In-hospital treatment**	
Intravenous thrombolysis	30 (19.7)
Dual antiplatelet therapy	109 (71.7)
Early neurological deterioration	24 (15.8)
3-month death	3 (2.0)
3-month unfavorable outcome	10 (6.6)

Data are number (%), mean ± SD, or median and IQR.

SD, standardized difference; IQR, interquartile range; ND, neurological deterioration; NIHSS, National Institutes of Health Stroke Scale; SBP, systolic blood pressure; DBP, diastolic blood pressure; TOAST, Trial of Org 10172 in Acute Stroke Treatment; TIA, transient ischemic stroke; ICH, intracerebral hemorrhage; NfL, neurofilament light chain; TC, total cholesterol; TG, triglycerides; LDL-C, low-density lipoprotein cholesterol; HDL-C, high-density lipoprotein cholesterol; CRP, C-reactive protein.

### Baseline characteristics and clinical outcomes between patients with MIS with and without END

Baseline characteristics and clinical outcomes were compared between patients with MIS with and without END (Table 2). The median sNfL levels of enrolled patients with MIS were significantly higher than the levels of 40 age- and sex-matched healthy controls (patients with MIS: median 63.1; IQR: 51.2–83.4 pg/ml; healthy controls median: 47.6; IQR: 40.8–56.1 pg/ml; *p* < 0.001), while patients with MIS with END had a higher level of sNfL compared with those patients without END (with END median: 74.1; IQR: 59.5–89.8; without END median: 61.2, IQR: 50.5–82.2; *p* = 0.026) (Figure 3). Meanwhile, the END group had a higher systolic blood pressure level on admission (168.1 ± 28.5 vs. 156.0 ±2 4.5 mmHg, p = 0.033) and less frequently received dual antiplatelet therapy (54.2 vs. 75.0%, *p* = 0.038). There was no difference in the age, sex, onset to admission time, baseline NIHSS score, diastolic blood pressure, vascular risk factors, stroke etiology, moderate–severe WML (defined as Fazekas' rating scale score 2–3), and other laboratory values between the two groups (all *p* > 0.05). Although there was no difference in the 3-month death between the two groups, the incidence rate of 3-month unfavorable outcome was significantly higher in patients with MIS with END (with END: 25.0%; without END: 3.1%; *p* < 0.001). After adjusting for age, sex, and baseline NIHSS score, END was still associated with an increased risk of the unfavorable outcome at 3 months [odds ratio (OR) 12.4, 95 % confidence interval (CI) 2.7 to 56.3, *p* = 0.001].

**Table 2:** Baseline characteristics and clinical outcomes between patients with MIS with and without END.

	**MIS with END (*n* = 24)**	**MIS without END (*n* = 128)**	***P-*value**
Age, years	68.3 ± 11.8	67.6 ± 11.1	0.755^*^
Sex (male)	14 (58.3)	90 (70.3)	0.247^‡^
Onset to admission time, hours	10.5 (3.8–24.0)	7.0 (3.0–18.3)	0.174^†^
Baseline NIHSS score	2 (2–3)	2 (1–3)	0.481^†^
SBP on admission (mm Hg)	168.1 ± 28.5	156.0 ± 24.5	0.033^*^
DBP on admission (mm Hg)	92.0 ± 17.9	88.1 ± 13.6	0.219^*^
Baseline serum glucose (mmol/L)	8.3 (5.9–13.1)	7.2 (6.0–10.4)	0.511^†^
**Risk factors**			
Hypertension	23 (95.8)	110 (85.9)	0.313^‡^
Diabetes mellitus	12 (50.0)	42 (32.8)	0.106^‡^
Dyslipidemia	7 (29.2)	45 (35.2)	0.570^‡^
Coronary heart disease	2 (8.3)	14 (10.9)	0.985^‡^
Atrial fibrillation	4 (16.7)	21 (16.4)	1.000^‡^
Rheumatic heart disease	1 (4.2)	3 (2.3)	0.501^#^
Gout	2 (8.3)	4 (3.1)	0.240^#^
Current smoking	11 (45.8)	72 (56.3)	0.347^‡^
Previous ischemic stroke/TIA	2 (8.3)	22 (17.2)	0.432^‡^
Previous ICH	1 (4.2)	5 (3.9)	1.000^#^
**TOAST classification**			0.624^#^
Large-artery atherosclerosis	10 (41.7)	39 (30.5)	
Cardio-embolism	1 (4.2)	15 (11.7)	
Small vessel occlusion	12 (50.0)	58 (45.2)	
Other determined etiology	0 (0)	2 (1.6)	
Undetermined etiology	1 (4.2)	14 (10.9)	
Fazekas' rating scale (score 2–3)	11 (45.8)	41 (32.0)	0.191^‡^
**Laboratory values**			
Serum NfL (pg/ml)	74.1 (59.5-89.8)	61.2 (50.5-82.2)	0.026^†^
TG (mmol/L)	1.3 (0.9-1.9)	1.4 (1.0-2.0)	0.531^†^
TC (mmol/L)	4.4 ± 1.0	4.5 ± 0.9	0.500^*^
LDL-C (mmol/L)	2.4 ± 0.8	2.5 ± 0.6	0.716^*^
HDL-C (mmol/L)	1.2 ± 0.3	1.3 ± 0.3	0.219^*^
Uric acid (μmol/L)	355.0 (304.1–428.1)	359.2 (279.7–417.1)	0.455^†^
Fibrinogen (mg/dL)	2.4 (2.1–2.7)	2.4 (2.2–3.0)	0.991^†^
D-dimer (mg/dL)	0.39 (0.22–0.63)	0.38 (0.23–0.83)	0.953^†^
CRP (mg/L)	0 (0–2.36)	0 (0–1.88)	0.722^†^
**In-hospital treatment**			
Intravenous thrombolysis	6 (25.0)	24 (18.8)	0.670^‡^
Dual antiplatelet therapy	13 (54.2)	96 (75.0)	0.038^‡^
3-month death	1 (4.2)	2 (1.6)	0.405^#^
3-month unfavorable outcome	6 (25.0)	4 (3.1)	< 0.001^‡^

Data are number (%), mean ± SD, or median and IQR.

SD, standardized difference; IQR, interquartile range; ND, neurological deterioration; NIHSS, National Institutes of Health Stroke Scale; SBP, systolic blood pressure; DBP, diastolic blood pressure; TOAST, Trial of Org 10172 in Acute Stroke Treatment; TIA, transient ischemic stroke; ICH, intracerebral hemorrhage; NfL, neurofilament light chain; TC, total cholesterol; TG, triglycerides; LDL-C, low-density lipoprotein cholesterol; HDL-C, high-density lipoprotein cholesterol; CRP, C-reactive protein.

* Student's *t*-test.

† Mann–Whitney *U*-test.

‡ χ^2^ test.

# Fisher's exact test.

**Figure 3:**
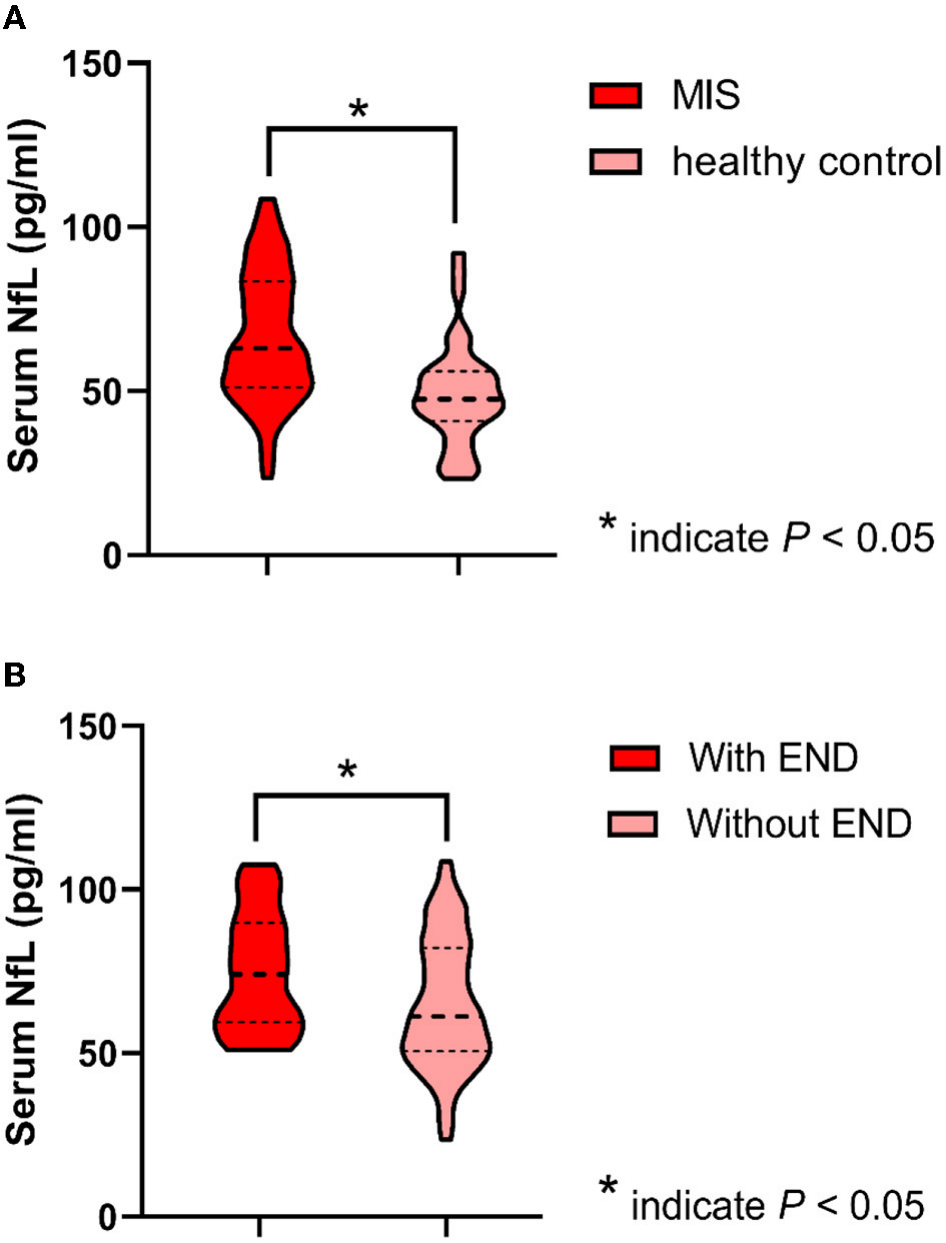
Serum neurofilament light chain (sNfL) levels between groups. **(A)** sNfL levels between patients with minor ischemic stroke (MIS) and healthy controls are shown as violin plots (patients with MIS median: 63.1, IQR: 51.2–83.4 pg/ml vs. healthy controls median: 47.6, IQR: 40.8–56.1 pg/ml; *p* < 0.001). **(B)** sNfL levels between patients with MIS with END and without END are shown as violin plots (with END median: 74.1, IQR: 59.5–89.8 pg/ml vs. without END median: 61.2, IQR: 50.5–82.2 pg/ml; *p* = 0.026).

### Multivariate analyses for the association between sNfL and END in patients with MIS

Variables that potentially affect END in patients with MIS (*p* < 0.1) were included in multivariate logistic regression analyses; the results are shown in Table 3. After adjusting for the baseline NIHSS score and potential confounding factors (Model 1), an elevated sNfL level (per 10 pg/mL) was associated with an increased risk of END (OR 1.36, 95% CI 1.04–0.77; *p* = 0.026) in multivariate analyses. When age was included in the multivariate logistic regression (Model 2), an elevated sNfL level (per 10 pg/mL) remained an independent risk factor for END (OR 1.35, 95% CI 1.04–1.77; *p* = 0.027). Moreover, systolic blood pressure on admission (OR 1.25, 95% CI 1.03–1.51) and dual antiplatelet therapy (OR 0.22, 95% CI 0.08–0.63) were independently associated with END in patients with MIS in the two multivariate logistic regression models (both with *p* < 0.05). An increased baseline NIHSS score also tended to be associated with a higher risk of END (OR 1.66, 95% CI 0.93–2.99; *p* = 0.090).

**Table 3:** Multivariate analyses for factors associated with END in patients with MIS.

	**Model 1**		**Model 2**	
**Variables**	**OR (95%CI)**	***P-*** **value**	**OR (95%CI)**	***P-*** **value**
Serum NfL, per 10pg/ml	1.36 (1.04–1.77)	**0.026**	1.35 (1.04–1.77)	**0.027**
SBP on admission, per 10 mmHg	1.25 (1.03–1.51)	**0.024**	1.25 (1.03–1.51)	**0.023**
Dual antiplatelet therapy	0.22 (0.08–0.63)	**0.005**	0.22 (0.08–0.63)	**0.005**
Age, year	–	–	1.00 (0.97–1.05)	0.840
Baseline NIHSS score	1.67 (0.93–3.00)	0.089	1.66 (0.93–2.99)	0.090

Model 1: Adjusted for the baseline NIHSS score and variables that had a potential association with early neurological deterioration in univariate analysis (*P* < 0.05) using the forward LR method and Hosmer and Lemeshow test (*P* = 0.867).

Model 2: Adjusted for age, baseline NIHSS score, and variables that had a potential association with early neurological deterioration in univariate analysis (*P* < 0.05) using the forward LR method and Hosmer and Lemeshow test (*P* = 0.865).

OR, odds ratio; CI, confidence interval; NfL, neurofilament light chain; NIHSS, National Institutes of Health Stroke Scale; SBP, systolic blood pressure.

### Stratified analyses and interaction tests to identify factors that might modify the association between sNfL and END

Stratified analyses and interaction tests were further employed to explore the association between sNfL levels and END in patients with MIS. Stratified logistic regression analyses demonstrated that the association between sNfL and END did not change by age group, sex, baseline NIHSS score, Fazekas' rating scale, hypertension, diabetes mellitus, intravenous thrombolysis, and dual antiplatelet therapy in patients with MIS (all *p* > 0.05) (Table 4).

**Table 4:** Stratified logistic regression analyses to identify variables that might modify the association between sNfL and END in patients with MIS.

Variable^*^	**Adjusted OR (95%CI)**	***P*-value**	***P* for interaction**
**Age**			0.334
< 70	1.6 (1.1–2.5)	0.026	
≥70	1.1 (0.7–1.6)	0.605	
**Sex**			0.463
Male	1.3 (0.9–1.9)	0.201	
Female	1.4 (0.9–2.0)	0.112	
**Baseline NIHSS score**			0.999
Score 0	Reference		
Score 1	1.3 (0.6–2.9)	0.445	
Score 2	1.4 (1.0–2.0)	0.074	
Score 3	1.3 (0.7–2.2)	0.414	
**Fazekas' rating scale**			0.657
Score 0–1	1.4 (1.0–2.0)	0.080	
Score 2–3	1.2 (0.8–1.9)	0.365	
**Hypertension**			0.933
Yes	1.4 (1.0–1.8)	0.022	
No	1.3 (0.5–3.7)	0.596	
**Diabetes mellitus**			0.777
Yes	1.3 (0.9–2.0)	0.190	
No	1.4 (0.9–2.1)	0.097	
**Intravenous thrombolysis**			0.866
Yes	1.3 (0.7–2.6)	0.446	
No	1.4 (1.0–1.9)	0.026	
**Dual antiplatelet therapy**			0.953
Yes	1.4 (1.0–1.9)	0.042	
No	1.3 (0.8–2.2)	0.340	

* Stratified logistic regression analysis to identify variables that modify the correlation between serum NfL (per 10 pg/ml) and END. Each stratification is adjusted for age group, sex, baseline NIHSS score, Fazekas' rating scale, hypertension, diabetes mellitus, intravenous thrombolysis, and dual antiplatelet therapy, except for the stratification factor itself.

OR, odds ratio; CI, confidence intervals; NIHSS, National Institutes of Health Stroke Scale.

## Discussion

Epidemiological studies showed that there are approximately 3 million new-onset strokes every year in China and approximately 1 million are MIS (29, 30). Since the baseline NIHSS score can strongly predict outcomes after stroke, the outcomes for patients with MIS are generally favorable (2). Yet, prospective data suggest that 4.5–26.4% of patients with MIS are also affected by early neurological worsening and become disabled (3, 27, 31, 32). The incidence rate of END in patients with MIS is 15.8% in our cohort. Differences in the incidence rate of END in patients with MIS may reflect heterogeneity in demographics (age, sex, and ethnicity) of the enrolled patients, the definition of MIS, and the way END was defined and measured, highlighting the need for a standardized definition of MIS and END. The median time from the stroke onset to the development of END in our cohort was 48 h (IQR: 30–49.75 h), similar to previous studies (3, 27, 32). Although the association between END and the outcome of patients with MIS remains to be established, our study suggested that MIS patients with END had a significantly higher rate of the 3-month unfavorable outcome than those without (25.0 vs. 3.1%). END was also associated with an increased risk of a 3-month unfavorable outcome in multivariate analysis after adjusting for age, sex, and baseline NIHSS score, as found in many other studies conducted in patients with ischemic stroke (4–6, 33, 34) and is consistent with our results. Until recently, the mechanisms underlying END in patients with MIS are still unclear, and no consensus has been reached on the risk factors of END (8, 15). Understanding the mechanisms underlying END in patients with MIS could provide valuable insights for rational prevention of END in patients with MIS. Moreover, the early targeting of patients at higher risk of END is of great importance for improving the outcome of MIS.

In a previous study, NfL was shown to be higher in patients with ischemic stroke than in healthy controls, whereas NfL in patients with transient ischemic stroke (TIA) was comparable to those in healthy controls (35). In patients with ischemic stroke, NfL levels have been correlated with initial stroke severity (11–14). In the present study, we found that the sNfL levels of patients with MIS were significantly higher than that of age- and sex-matched healthy controls, while patients with MIS with END had a higher level of sNfL compared with those patients without END. Although it has been demonstrated that the baseline NIHSS score could strongly predict the course of END (15–17), multivariate analyses adjusting for confounders, including age and baseline NIHSS score, also suggested that an elevated sNfL level was independently associated with END in patients with MIS. Stratified analyses and interaction tests demonstrated that the association between sNfL and END did not change by age group, sex, baseline NIHSS score, Fazekas' rating scale, hypertension, diabetes mellitus, intravenous thrombolysis, and dual antiplatelet therapy in patients with MIS. Some studies have suggested an association between baseline sNfL levels and final infarct size on MRI (13, 26, 36), but others have reached a different conclusion (11, 12). Therefore, our results could not be explained by the effect of final infarct volume on the sNfL levels. Neuronal damage and loss are the pathological substrates of disability caused by an AIS. As a part of the neuronal cytoskeleton that is exclusively expressed in neurons, neurofilaments are suitable candidate biomarkers of ischemic neuronal injury (9, 37). It has been demonstrated that sNfL levels increased during the first few days after the stroke onset and remained increased over 3–6 months (26, 36). Experimental studies suggest that synaptic NfL plays an essential role in controlling synaptic function, neurotransmission, and stabilizing NMDA receptors in the neuronal cell membrane (38–40). According to the currently available evidence, elevated sNfL levels after AIS seem to reflect the extent of neuronal injury, persistent blood–brain barrier breakdown, and ongoing post-ischemic immunological or inflammatory processes. Meanwhile, elevated sNfL levels may act as a biomarker of neural plasticity and a positive predictor of functional improvement (9, 41). All these findings suggest a potential molecular mechanism that links the sNfL with the risk of neurologic worsening and functional disability. The present study is the first to report a positive correlation between sNfL levels and END in Chinese patients with MIS. Therefore, sNfL might be a promising biomarker candidate that can help identify MIS patients at high risk of END, for reaching individual therapeutic decisions in clinical trials. Further studies with large sample sizes are needed to determine the optimal cutoff value of sNfL as an indicator for END and validate sNfL as a biomarker for END in patients with MIS.

It is also worth noting that dual antiplatelet therapy was associated with decreased risk of END in patients with MIS in our cohort (OR 0.22, 95% CI 0.08–0.63), which are in line with previous studies conducted in patients with AIS (42–44). In addition, higher baseline systolic blood pressure (OR 1.25, 95% CI 1.03–1.51) was associated with increased END risk in patients with MIS. These results support that platelet aggregation might be an important mechanism of END (45). Our results also support the view that an impaired cerebral hemodynamic response due to hypertension might be another contributor to END (45). Therefore, dual antiplatelet therapy in the acute phase of MIS and premorbid personalized antihypertensive treatment may potentially reduce END and subsequently improve the outcome of patients with MIS. However, the present study was not specifically targeted at this treatment effect, thus, our results should be interpreted cautiously. Further studies targeting patients with MIS at high risk of END are warranted to determine the usefulness of different acute therapy strategies.

### Limitations

The results of the present study should be interpreted with caution, given its limitations. First, it was a single hospital-based study conducted in China, with limited generalizability. Second, the sample size of our study was relatively small, and only 24 cases suffered END. We could not determine the optimal cutoff value of sNfL as an indicator for END in patients with MIS. Third, sNfL levels may change dynamically after acute ischemic stroke. In the present study, sNfL levels were tested one time on admission. We did not have longitudinal data on sNfL levels. Further studies are needed to evaluate the association between dynamic changes in sNfL levels and END in patients with MIS. In addition, the number and location of the infarcts were not assessed in MRI imaging, as well as the infarct volume, which might have an association with END in patients with ischemic stroke. Meanwhile, the renal function might affect the level of sNfL. However, due to a lack of data, we did not include the eGFR levels in the multivariate analyses. In addition, although non-neurological complications, such as infections, might cause clinical deterioration and an increase in the NIHSS score by causing a confusional state and decreasing the level of consciousness, we did not include medical complications in the analyses because of a lack of data. Moreover, we performed the follow-up by telephone interview or a mailed questionnaire instead of a clinic visit which may result in reporting bias. Finally, our study was an observational study. No causal link could be drawn. Thus, well-designed studies with large sample sizes are needed to validate our findings in the future.

## Conclusion

We conducted a prospective observational study in Chinese patients with acute MIS admitted within 24 h from the symptom onset. We identified that END is common in patients with MIS and associated with 3-month unfavorable outcomes. An elevated sNfL level was independently associated with END in patients with MIS. sNfL might be a promising biomarker candidate that can help identify patients with MIS at high risk of END, for reaching individual therapeutic decisions in clinical practice.

## Data availability statement

The raw data supporting the conclusions of this article will be made available by the authors, without undue reservation.

## Ethics statement

The studies involving human participants were reviewed and approved by Ethics Committee of Deyang People's Hospital (Reference No. 2019-01-142-K01). The patients/participants provided their written informed consent to participate in this study.

## Author contributions

JL and XY: conceived the study, analyzed and interpreted the data, as well as drafted the manuscript. HC and CW: contributed to study supervision. PZ, YZ, YD, SL, and JF: participated in data collection. JL and PZ: participated in statistical analysis, data interpretation, and revised the manuscript. All authors critically revised the manuscript for important intellectual content and approved the final manuscript.
