# Supplementary material for: Serum neurofilament light chain levels are associated with early neurological deterioration in minor ischemic stroke
Source: Front Neurol. 2023 Mar 9;14:1096358. doi: 10.3389/fneur.2023.1096358 (PMC10034185; doi:10.3389/fneur.2023.1096358)
Supplement: Supplementary file 1 [file Image_1.pdf]

Supplemental figure 1. Correlation between baseline NIHSS score and sNfL values at hospital admission.

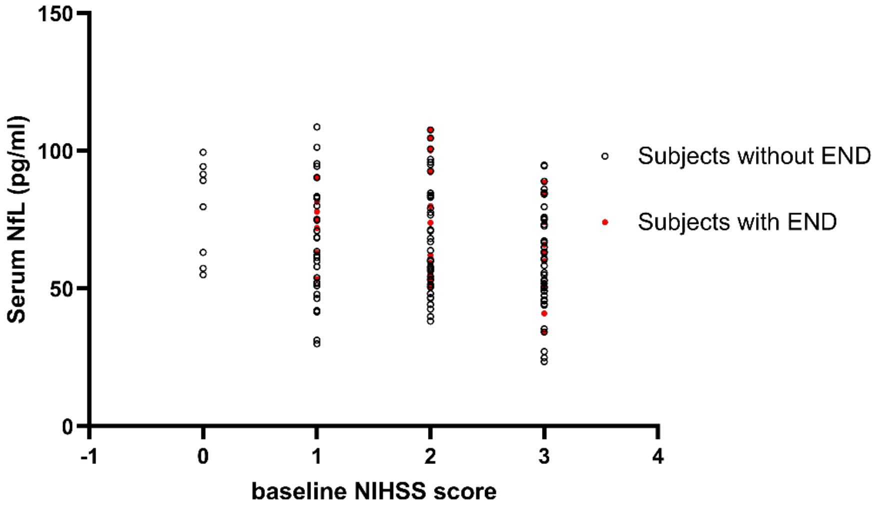

Correlation between baseline NIHSS score and sNfL values at hospital admission are shown a scatter plot (spearman correlation analysis, rho=0.150, p=0.066).
